# Effects of Vitamin B6 Deficiency on the Composition and Functional Potential of T Cell Populations

**DOI:** 10.1155/2017/2197975

**Published:** 2017-03-06

**Authors:** Bingjun Qian, Shanqi Shen, Jianhua Zhang, Pu Jing

**Affiliations:** ^1^Department of Nutrition and Health, Research Centre of Biomedical Technology Co. Ltd., Yancheng Vocational Institute of Health Sciences (YIHS), Jiangsu 224005, China; ^2^Research Center for Food Safety and Nutrition, Key Lab of Urban Agriculture (South), Bor S. Luh Food Safety Research Center, School of Agriculture & Biology, Shanghai Jiao Tong University, Shanghai 200240, China

## Abstract

The immune system is critical in preventing infection and cancer, and malnutrition can weaken different aspects of the immune system to undermine immunity. Previous studies suggested that vitamin B6 deficiency could decrease serum antibody production with concomitant increase in IL4 expression. However, evidence on whether vitamin B6 deficiency would impair immune cell differentiation, cytokines secretion, and signal molecule expression involved in JAK/STAT signaling pathway to regulate immune response remains largely unknown. The aim of this study is to investigate the effects of vitamin B6 deficiency on the immune system through analysis of T lymphocyte differentiation, IL-2, IL-4, and INF-*γ* secretion, and* SOCS-1* and* T-bet* gene transcription. We generated a vitamin B6-deficient mouse model via vitamin B6-depletion diet. The results showed that vitamin B6 deficiency retards growth, inhibits lymphocyte proliferation, and interferes with its differentiation. After ConA stimulation, vitamin B6 deficiency led to decrease in IL-2 and increase in IL-4 but had no influence on IFN-*γ*. Real-time PCR analysis showed that vitamin B6 deficiency downregulated* T-bet* and upregulated* SOCS-1* transcription. This study suggested that vitamin B6 deficiency influenced the immunity in organisms. Meanwhile, the appropriate supplement of vitamin B6 could benefit immunity of the organism.

## 1. Introduction

The immune system's role is crucial in prevention and control of pathogenic infection as well as various cancers [1]. Meanwhile, the natural aging process, malnutrition, and increased stress brought upon by the fast-paced urban lifestyle have been demonstrated to decrease immunity [2–4]. Among these factors, the effect of malnutrition on immunity has been widely investigated on children in developing countries, people with eating-disorder problems, and the elderly [5–7]. Close to one billion people suffer from varying degrees of malnutrition as a result of insufficient food or food lack of micronutrients [4]. Vitamin B6 deficiency is a very common phenomenon, especially among women of childbearing age as well as the elderly [8–10].

Vitamin B6 is an intriguing micronutrient that mediates numerous metabolic processes in vivo including amino acid metabolism, gluconeogenesis, lipid metabolism, and nervous system development and functioning. Vitamin B6 has been implicated in the regulation of immune responses that are associated with a wide range of diseases, including inflammation [11] and various cancers [1, 12, 13]. Previous studies suggested that vitamin B6 deficiency could impair immune responses. Kumar and Axelrod found that the serum antibody (IgG, IgM) production in vitamin B6 deficient mice decreased after goat erythrocyte immune stimulation and could be recovered to normal level after a short-term of vitamin B6 supplementation [14]. Doke et al. found that the vitamin B6 deficient mice produces a specific IgE antibody compared to normal controls after the dinitrophenylated ovalbumin (DNP-OVA) immune stimulation [15]. The level of IL-4, an essential factor for IgE synthesis, was significantly higher in the vitamin B6 deficient mice than in the normal controls, while the level of IL-2 in deficient groups was significantly lower than in the normal controls [15]. Subsequently, vitamin B6 supplementation in the deficient groups resulted in the serum antibody (IgG) and anti-DNP IgE recovering to the same levels as those in the normal controls [15]. Further research showed that excessive vitamin B6 (6 mg/100 g diet) supplementation could inhibit the production of the anti-OVA antibody IgE and IgG1 due to the suppression of hepatic cathepsin B activity by vitamin B6 [16]. Therefore, a moderation of vitamin B6 might mediate immune signal transduction or regulate immune cell differentiation and cytokine production together with other signal molecules to reach an immune homeostasis.

Janus tyrosine kinase/signal transducer and activator of transcription (JAK/STAT) signaling pathway exists in almost all cytokine signaling pathways [17]. Many intracellular cytokines, including the colony-stimulating factor, interleukins (ILs), interferons (IFNs), erythropoietin (Epo), and thrombopoietin (Tpo), can bind with type I or II cytokine receptors to transduce the signal into the nucleus via the JAK/STAT signaling pathway [18]. These cytokines can further induce the expression of downstream genes and regulate a series of biological effects including immune response and cell growth [18]. Suppressor of cytokine signaling (SOCS) is a cytokine signal transduction suppressor in the JAK/STAT signaling pathway [19]. SOCS-1 could inhibit the differentiation of the IFN-*γ*-expression in cells or terminate IFN-*γ* signal transduction to block the signaling [20]. The expression of IL-1b, IL-2, and IL-2R was suppressed in CD4-lymphocytes in the vitamin B6 deficient mice [21] due to decreased serine hydroxymethyl transferase (SHMT) activity in the absence of vitamin B6, resulting in the reduction of one carbon unit and the blocking of mRNA synthesis, which in turn affects the gene expression. However, IL-4 expression increased in vitamin B6 deficient mice [15]. This was opposite to the decline of gene expression caused by the reduction of one carbon unit.

Therefore, this study mainly investigated the effect of vitamin B6 deficiency on the composition and functional potential of T cell populations through analysis of pyridoxine 5′-phosphate (PLP) and xanthurenic acid (XA) plasma level, lymphocyte proliferation and differentiation, cytokines expression, and* SOCS-1* and* T-bet* transcription.

## 2. Materials and Methods

### 2.1. Materials and Chemicals

The male BALB/c mice were purchased from Shanghai Slac Laboratory Animal Center. The diet contained sugar, fiber, salt, and oil that were all food-grade and purchased from Auchan supermarket (Shanghai, China). The micronutrients, such as vitamins A, D, E, B1, B2, B6, B12, and K1 and folic acid, were provided by Shanghai Xinyi Medical Co. Ltd. (Shanghai, China). Nicotinic acid and pantothenic acid were purchased from the Generay Biotech (Shanghai, China). Selenium and Biotin were from Swanson (Fargo, ND, USA). Other chemicals were all chemically pure and provided by Sinopharm Chemical Reagent (Shanghai, China). Deionized water was supplied as the drink for mice.

### 2.2. Ethics Considerations

Animal study was carried out strictly in accordance with the Guidelines for the Care and Use of Laboratory Animals of Shanghai Jiao Tong University. The protocol was approved by Shanghai Municipal Laboratory Animal Management Office, Shanghai Municipal Science and Technology Commission (Permit Number: 11ZR1416200).

### 2.3. Construction of Vitamin B6 Deficient Mouse Model

In order to investigate the influence of vitamin B6 deficiency on the immune system and the subsequent remediation by vitamin B6 supplementation, three-week-old BALB∖c mice, weighing an average of 10 g, were randomly divided into 4 dietary groups, namely, the control group (+VB6+), the deficiency group (−VB6−), the recovery group (−VB6+), and the excess group (−VB6++). Each group contained 7 mice. They were housed in the standard cages with a 12 h light/12 h dark cycle. The temperature and humidity in the cages were controlled at 24 ± 1°C and 60 ± 5%, respectively.

The diet formula (Table 1) was based on the previous studies by Miller & Baumann [22], Doke et al. [15], and Inubushi et al. [16]. Mineral substances and vitamin demands were based on the standard “Laboratory Animal-Nutrients for Formula Feed” (GB14924.3-2010, China). The vitamins listed in Table 1 were ground into powders and mixed with other diet ingredients to form a daily diet. All the diets contained the same composition, except vitamin B6. The diet was strictly defined as Non-VB6 Diet, Normal-VB6 Diet, and Excessive-VB6 Diet, which contain 0, 12 mg, and 120 mg of vitamin B6 per kg diets, respectively. The Normal-VB6 Diet contained the daily recommended intake (DRI) of vitamin B6 (12 mg), while the Excessive-VB6 Diet contained 10 times the vitamin B6 in the Normal-VB6 Diet.

The control group (+VB6+) was fed the Normal-VB6 Diet throughout the experiment, and the other three groups were deprived of vitamin B6 by being fed with Non-VB6 Diet for the first 5 weeks. Following this, the deficiency group (−VB6−) continued to take the Non-VB6 Diet, and the recovery group (−VB6+) was changed to the Normal-VB6 Diet, while the excess group (−VB6++) took the Excessive-VB6 Diet until the end of the experiment. Diet and water were given ad libitum. Water contained the same composition of vitamins as the diet.

### 2.4. Weight Measurement

To estimate the effect of vitamin B6 deficiency on growth, the body weight of the mice was measured weekly. A basic growth curve of the mice was constructed and the differences among the 4 groups were comparatively analyzed.

### 2.5. Determination of Xanthurenic Acid and Pyridoxal 5′-Phosphate in Plasma by LC-MS/MS

To confirm the success of the vitamin B6 deficient mouse model, XA (a metabolic intermediate of tryptophan) and PLP (the active form of vitamin B6), plasma levels were assayed using ultra performance liquid chromatography (ACQUITY UPLC system, MA, USA) linked with a triple quadrupole mass spectrometer of AB SCIEX Triple Quad™ 5500 LC-MS/MS System (AB SCIEX, Toronto, Ontario, Canada) in the Instrumental Analysis Center of Shanghai Jiao Tong University. Blood samples were treated according to the description by Midttun et al. [23]. Briefly, orbital blood of mice was collected into tubes, which were heparinized for 1 day prior to scarification to prevent coagulation. Then, plasma was carefully pipetted into another microtube on ice after being centrifuged at 2000*g* for 10 min at 4°C and stored at −80°C. Sixty microliters of blood plasma was deproteinized for 60 min by adding an equal volume of 60 g/L ice-cold trichloroacetic acid (TCA), followed by a centrifugation at 5800*g* for 15 min at 4°C to remove the denatured protein. The supernatant (60 *μ*L) was removed to a new tube to be dried by blowing gaseous nitrogen. Finally, methanol was added to a final volume of 200 *μ*L to dissolve the treated sample. A 100 ng/mL mixture of XA and PLP (Sigma, St. Louis, MO, USA) was used as standard, respectively. A Zorbax stable-bond C8 reversed-phase column (80 Å, 3.5 *μ*m, 150 × 4.6 mm; Agilent) equipped with a similar guard column (80 Å, 5 *μ*m, 12.5 × 4.6 mm; Agilent) was used with a mobile phase consisting of solution A (650 mmol/L acetic acid), solution B (100 mmol/L heptafluorobutyric acid in A), and solution C (90% acetonitrile in water) at a flow rate of 1.3 mL/min. XA and PLP were identified on the basis of *m*/*z* ratio and retention time. There was no cross-talk between ion pairs from those different analytes. Quantification was by a linearity gradient curve; *R*^2^ was 0.9988 (0.304–100 ng/mL) and 0.9975 (0.304–100 ng/mL), respectively, for XA and PLP.

### 2.6. Total Spleen Lymphocytes Isolation

Total spleen lymphocytes were isolated for further cellular experiments. Mouse spleen lymphocytes were isolated from the 4 groups of mice by using a standard protocol [24] with the Cappel LSM lymphocyte separation medium (MP Biomedicals Solon, OH, USA). The mice were sacrificed via euthanasia and placed into 70% ethanol for disinfection. The spleen was removed quickly with an aseptic technique and washed thoroughly with RPMI-1640 medium (Gibco, Invitrogen, Carlsbad, CA, USA). The spleen cells were then released by being ground on 200 mesh grids and resuspended in 5 mL RMPI-1640 medium. The cells were then transferred to a 15 mL tube and an equal volume of LSM was added. After centrifuging at 1300*g* for 20 min with slow acceleration and slow deceleration, the middle layer of spleen lymphocytes was pipetted carefully and resuspended in RPMI-1640 medium supplemented with 10% fetal bovine serum (FBS) (Biological Industries, Beit HaEmek, Israel). Spleen lymphocytes count and viability were assayed by trypan blue prior to final plating. The cell concentration was adjusted to 1 × 10^6^/mL. The suspension was then seeded into 96-well plates and incubated at 37°C in a humidified atmosphere (5% CO_2_).

After 2 h incubation, half of the murine spleen lymphocytes were incubated with 5 *μ*g/mL concanavalin A (ConA, type IV; Sigma, St. Louis, MO, USA) to stimulate immune reactions, such as T lymphocyte differentiation, proliferation and cytokine production, and immune related gene mRNA transcription. Cells without ConA stimulation were set as control, where equal volume of RMPI-1640 medium was added as substitute.

### 2.7. Lymphocyte Proliferation Assay

To estimate the effect of different doses of vitamin B6 on immunomodulatory potential, spleen lymphocyte proliferation was assessed using a WST-8 Cell Counting Kit-8 (Beyotime, shanghai, China) after 72-hour stimulation with ConA. Ten microliters of CCK-8 solution was added to each well away from light and incubated for another 2 h under the same conditions. The absorbance at 450 nm was determined by the Multiskan GO Microplate spectrophotometer (Thermo, USA) with three technical repeats. Proliferation response was expressed as stimulation index (SI) calculated as the ratio of the mean OD_450_ value of the ConA-stimulated cells to the mean OD_450_ value of the medium alone-stimulated cells.

### 2.8. T Lymphocyte Differentiation Assay

To investigate the trend of T lymphocyte differentiation influenced by the different doses of vitamin B6 in diet after immune stimulation, the proliferation of the CD3+ cells was estimated to represent that of total T lymphocytes, and percentages of two main T- lymphocyte cell subsets associated with immunomodulation, CD4+ (helper T lymphocytes) and CD8+ (cytotoxic T lymphocytes), were estimated by detecting their cell surface specific glycoproteins CD4 and CD8 molecule, respectively, using an ELISA assay kit (Shanghai XinRan Biological, Shanghai, China) according to Franke et al.'s description [25] with some modifications. All kit reagents were allowed to reach room temperature prior to usage. The absorbance was determined at 450 nm by the Microplate spectrophotometer (Multiskan GO; Thermo Scientific, Waltham, MA, USA). The standard curves of CD3, CD4, and CD8 were established by plotting the U/mL concentrations versus absorbance values of the standard wells. The curves were used to quantify the concentration of CD3, CD4, and CD8 in cell culture supernates.

### 2.9. Cytokine IL-2, IL-4, and IFN-*γ* Secretion Levels

To investigate the secretion of cytokines related to immune regulation, such as interleukin-2 (IL-2), interleukin-4 (IL-4), and interferon-*γ* (IFN-*γ*), mouse IL-2/IL-4/IFN-*γ* ELISA sets (eBioscience, San Diego, CA, USA) were used to measure the IL-2, IL-4, and IFN-*γ* production following the manufacturer's recommendation. Each experiment was performance with three technical repeats.

### 2.10. Analysis of SOCS-1 and* T-bet* Gene Transcriptional Level by Real-Time PCR

To estimate the transcriptional levels of* SOCS-1 *and* T-bet *genes involved in the JAK/STAT immunomodulation signaling pathway, total RNA was extracted from the T lymphocyte from 4 groups of mice with or without ConA stimulation using the RNeasy Mini Kit (Qiagen, Santa Clarita, CA, USA) by following the manufacturer's recommendation. The ReverTra Ace-a-First-Strand cDNA synthesis kit (Toyobo, Japan) was employed to synthesize the oligo (dT) primed first-strand cDNA with 0.3 mg RNA as templates. Quantitative real-time-PCR (qRT-PCR) analysis was performed using SYBR Premix EX Taq (TaKaRa, Japan) on a Bio-Rad CFX96 Touch™ Real-Time PCR Detection System (BioRad, Hercules, CA) as described by Ding et al. [26] with the exception of the annealing temperature at 58°C when using the primers: SOCS-1 (forward: 5′-TCCGATTACCGGCGCATCACG-3′, reverse: 5′-CTCCAGCAGCTCGAAAAGGCA-3′) and T-bet (forward: 5′-GCCAGGGAACCGCTTATATG-3′, reverse: 5′-GACGATCATCTGGGTCACATTGT-3′). Gene* GAPDH* was used as reference gene with primers GAPDH (forward: 5′-CCATGGAGAAGGTGGG-3′, reverse: 5′-CAAAGTTGTCATGGATGACC-3′). Three biological replicates were used, each with three technical repeats.

### 2.11. Statistical Analysis

Seven replications with three independent experiments were performed. Data was reported as mean ± SEM or SD. One-way ANOVA and LSD test at the level of 0.05 were used to identify differences in means. Correlations among the indices of vitamin B-6 status were analyzed using a two-tailed Pearson correlation coefficient. Statistics analyses were carried out using SPSS for Windows (version rel. 10.05, 1999, SPSS Inc., Chicago, IL, USA).

## 3. Results

### 3.1. Vitamin B6 Deficiency Retards Growth Rate in Mice

**Figure 1:**
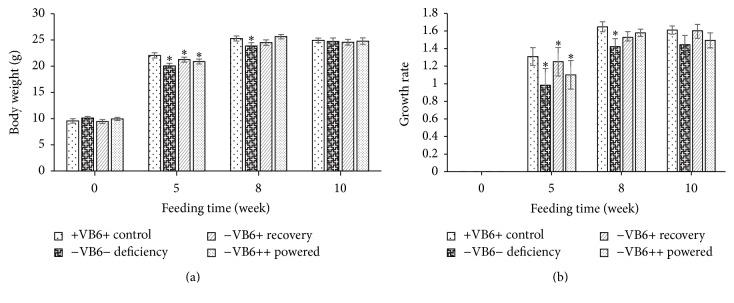
Body weight and body weight gain rate of BLAB/c mice fed with the experiment diets. Growth rate was calculated using the formula: Growth  rate = (Body weight_*n* week_ − Body weight_0  week_)/Body weight_0 week_. Each column represents the mean ± SEM (*n* = 7) with three independent experiments. Mean values with asterisk (*∗*) attached indicate significant difference (*P* < 0.05) among the groups at the same time point.

Previous studies showed that vitamin B6 deficiency could reduce body weight of organisms [15]. Four types of diets were formulated to investigate the effect of vitamin B6 deficiency and repletion on the growth of mice and their weight-for-age growth curve was established (Figure 1(a)). At the 5-week time point, the end of vitamin B6 deficiency, the vitamin B6 deficient diet-3 groups grew significantly slower than the control group (+VB+) (*P* < 0.05) (Figure 1(b)). At the 8-week time point, the vitamin B6 deficient group (−VB6−) still grew significantly slower than the other 3 groups (+VB6+, −VB6+, and −VB6++) (*P* < 0.05), among which there was no significant difference (*P* > 0.05).

### 3.2. Plasma Concentrations of PLP and XA

For further confirming vitamin B6 status in organisms, plasma levels of PLP and XA were analyzed by LC-MS/MS. In the −VB6− group, the plasma PLP level was not detected (Table 2). The plasma PLP levels in the other 3 groups increased following the order of −VB6++ > −VB6+ > +VB6+, suggesting that the mice could absorb and store vitamin B6 to compensate for the malnutrition caused by a 28-day depletion. However, the 120 mg vitamin B6/kg diet intake did not improve the plasma PLP concentration 10 times more than 12 mg/kg diet vitamin B6 intake. It might be due to the fact that 120 mg/kg diet of Vitamin B6 seriously exceeded the recommended dietary allowance (RDA) and the absorption of nutrients displayed saturation kinetics [27]. Meanwhile, the mean value of plasma XA levels in the −VB6− group (196.61 ng/mL) was significantly higher than that in +VB6+, −VB6+, and −VB6+ group (*P* < 0.05) (Table 2). Even though there was a certain amount of PLP in the plasma, XA was still detected, presenting the same tendency with Miller and Baumann's study [22]. The subsequent urinary XA level began to reduce when changing to vitamin B6-repletion diet and reached the lowest point after 40 days of recovery feeding, suggesting that the recovery from the dysfunction caused by vitamin B6 deficiency takes time.

### 3.3. Vitamin B6 Deficiency Weak Capacity of Lymphocyte Proliferation

**Figure 2:**
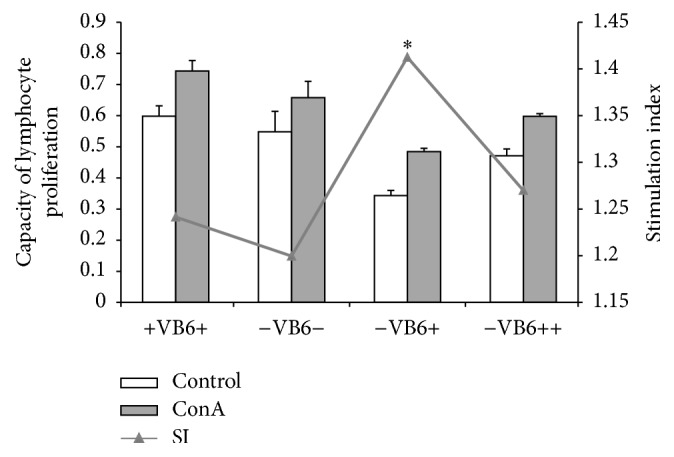
Capacity of lymphocyte proliferation. The stimulation index (SI) was calculated using the following formula “proliferation of T lymphocytes incubated with ConA stimulation divided by proliferation of T lymphocytes incubated without ConA stimulation.” Each column data represents mean values of seven replications with three independent experiments. SI index with *∗* denotes significant differences among the groups (*P* < 0.05).

The effect of vitamin B6 on the proliferation capacity of lymphocytes was estimated using the stimulation index (SI) via ConA stimulation. ConA was chosen for T lymphocytes stimulation, while PHA is used for B lymphocytes. The results showed that vitamin B6 deficiency slightly reduced the proliferation of lymphocytes by 3.37% compared with the +VB6+ group (Figure 2), although there were no significant differences in the SI among groups of +VB6+ (SI, 1.2415), −VB6− (SI, 1.1996), and −VB6++ (SI, 1.2700). After supplementation of normal dose of vitamin B6 (12 mg/kg diet, −VB6+ group) for 35 d, the proliferation of lymphocytes was recovered to 1.415 and was significantly stronger than that of the other three groups (*P* < 0.05). However, the excessive supplementation (120 mg/kg diet, −VB6++ group) did not significantly improve the lymphocyte proliferation (*P* > 0.05).

### 3.4. Vitamin B6 Deficiency Inhibits CD4 T Lymphocyte Differentiation after ConA Stimulation

**Figure 3:**
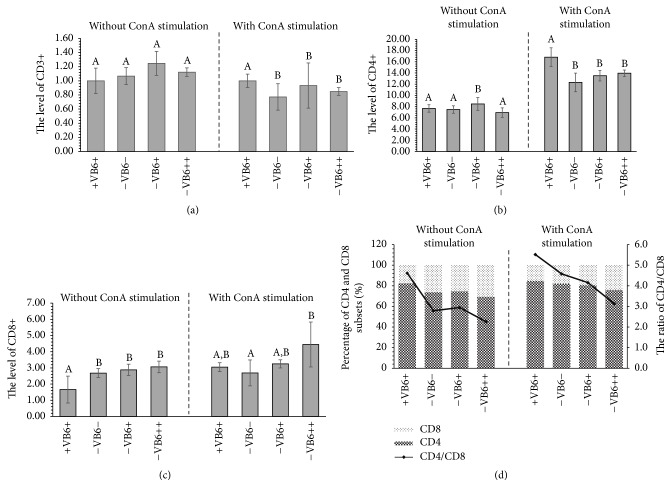
Level of T lymphocyte cell with or without ConA stimulation ((a), (b), and (c)) and differentiation of CD4+ and CD8+ subsets with or without ConA stimulation (d). Calculation was based on the percentage of concentration. Each group data represents mean values of seven replications with three independent experiments. Column marked with different alphabets denotes significant differences among the groups (*P* < 0.05).

The result showed that CD3+ cells representing total T lymphocytes in −VB6− group was not significantly different from the other 3 groups of −VB6+, +VB6+, and −VB6++ without ConA stimulation (Figure 3(a)), although it was less than −VB6+ and −VB6++ group. Moreover, without ConA stimulation, CD4+ and T-helper cell in the groups of −VB6− (73.63), −VB6+ (74.66), and −VB6++ (69.39) were all less than those of the group +VB6+ (82.20), and CD8+ and cytotoxic T lymphocyte in groups −VB6− (26.37), −VB6+ (25.34), and −VB6++ (30.64) were more than those in the group of +VB6+ (17.89) (Figures 3(b), 3(c), and 3(d)). Meanwhile, the ratio of CD4/CD8 in −VB6− group was significantly less than that in +VB6+ group (*P* < 0.05) (Figure 3(d)).

However, CD3 in −VB6− group decreased significantly compared with that in +VB6+ group after ConA stimulation (*P* < 0.05) (Figure 3(a)). In the −VB6+ and −VB6++ groups, the levels of CD3+ were more than that in −VB6− group but less than that in +VB6+ group (Figure 3(a)). Meanwhile, ratio of CD4/CD8 was decreased in −VB6−, −VB6+, and −VB6++ groups (Figure 3(d)), suggesting that the reduction of immunity was mainly contributed by the reduction of CD3 proliferation and the impairment of T cell differentiation caused by vitamin B6 deficiency.

### 3.5. Effect of Vitamin B6 Deficiency on Cytokines IL-2∖IL-4∖IFN-*γ* Secretion Level

**Figure 4:**
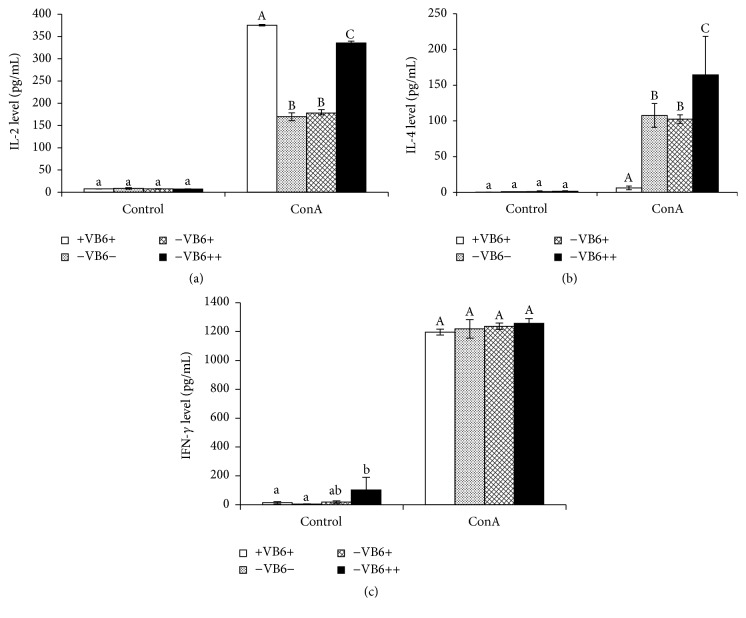
Cytokines IL-2 (a), IL-4 (b), and IFN-*γ* (c) secretion level of different vitamin B6 diet mice splenocytes after ConA stimulation. 5 *μ*g/mL ConA was added to stimulate the splenocyte, while the control received the same volume of RMPI1640 medium. After 48 h of incubation, cytokine levels in the culture medium were measured by ELISA method. Each column represents the mean ± SD of seven replications with three independent experiments. Columns marked with different letters are significantly different (*P* < 0.05) among different diet mouse.

Without ConA stimulation, IL-2 secretions of T lymphocytes from the four groups were all at a low level (Figure 4(a)). However, after ConA stimulation, IL-2 secretion levels in the −VB6+ group were higher than the −VB6− group, although the difference was not significant (*P* > 0.05). IL-2 secretion levels in the +VB6+ and −VB6+ groups were significantly higher than that of the other two groups (*P* < 0.05), but excessive vitamin B6 supplementation did not promote the IL-2 secretion much more than normal diet.

Without ConA stimulation, the IL-4 secretion levels of four groups were low. After ConA stimulation, IL-4 secretion levels significantly improved (*P* < 0.05) in the four groups and increased much more in −VB6− group than in +VB6+ and −VB6+ groups but less than in −VB6++ group (Figure 4(b)).

On the other hand, without ConA stimulation, IFN-*γ* production in the primitive spleen lymphocyte from the four groups was at low levels, although the −VB6++ group was higher than the other 3 groups (Figure 4(c)). After stimulation, IFN-*γ* increased significantly in all experiment groups and with no significant difference observed between the treatments (*P* > 0.05).

### 3.6. Down- and Upregulation of Gene SOCS-1 and* T-bet* Associated with JAK/STAT Signaling Pathway

**Figure 5:**
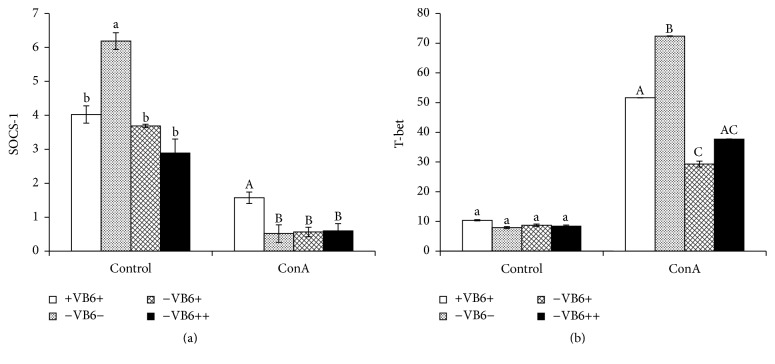
Real-time RNA results. mRNA SOCS-1 (a), T-bet (b) expression level of different vitamin B6 diet mice splenocytes after ConA stimulation. 5 *μ*g/mL ConA was added to stimulate the splenocyte, while the control received the same volume of RMPI1640 medium. After 24 h of incubation, mRNA was extracted and reversed. Each column represents the mean ± SD (*n* = 7) with three independent experiments. Columns marked with different letters are significantly different (*P* < 0.05) among different diet mouse.

The results of real-time PCR analysis showed that transcriptional levels of* SOCS-1* were higher in vitamin B6 deficient mice than in the other three treated mice groups (Figure 5(a)), consistent with the expression level of the IFN-*γ* expression (Figure 4(c)). After ConA stimulation, the transcriptional level of* SOCS-1* was significantly downregulated. There was no significant difference among −VB6−, −VB6+, and −VB6++ group (*P* > 0.05), while the transcriptional level of* SOCS-1* was significantly higher in +VB6+ group than in the other three treatment groups (*P* < 0.05) although no significant difference of IFN-*γ* level among these four groups was observed. Transcriptional levels of* T-bet* in the four groups were all low without ConA stimulation. However, it was upregulated after ConA stimulation and improved more significantly in the –VB6− group than in the −VB6+ and −VB6++ groups (*P* < 0.05), all of which were significantly higher than in +VB6+ group (*P* < 0.05) (Figure 5(b)).

### 3.7. Correlations between the Vitamin B6 Status and Immune Responses

Pearson correlation coefficients were performed to understand the relationship between the plasma PLP and immune response indicators (Table 3). Before ConA stimulation, plasma PLP levels were inversely associated with plasma XA level, CD4+ T lymphocyte numbers, IL-2 expression levels, and* SOCS1* transcriptional level but positively correlated with spleen lymphocyte proliferation, the ratio of CD4/CD8, and IL-4 and IFN-*γ* expression level. However, after ConA stimulation, plasma PLP concentration had positive correlation with IL-2 expression level, and* T-bet* transcription played a more important role than* SOCS-1 *transcription did. This also indicates that vitamin B6 plays an important role in immunomodulation.

## 4. Discussion

Vitamin B6 is an important micronutrient for our health, and its deficiency could weaken immunity [14–16], including decrease of the serum antibody and IL-2 production and increase of the level of IL-4.

We constructed a vitamin B6-deficient mouse model via vitamin B6-depletion diet and found that mice in −VB6− group grew significantly slower than the +VB6+ group (*P* < 0.05) at the 5-week time point, the end of vitamin B6 deficiency. In this study, water was supplied sufficiently and given ad libitum, so the loss of mouse weight was not affected by dehydration. Riordan et al., [28] also found that vitamin B6 deficiency reduces the rat growth to about 0.49 times the normal, possibly attributable to the reduced rats' appetite caused by vitamin B6 deficiency. In this study, we observed no statistical change in food consumption in vitamin B6 supplementation and deficiency groups, which indicated that the vitamin B6 deficiency might slow the growth of mice. Lewicka et al. [29] also demonstrated that vitamin B6 supplementation can statistically increase the average weight of rats fed with protein deficiency diets, a difference of 1.5 times in comparison to the nonsupplemented group after 30 days of study. Also, supplementation of vitamin B6 could help organisms recover some of the temporary weight loss caused by vitamin B6 deficiency, although, at the end of feeding, no significant difference in final weight among all groups was observed (*P* > 0.05), which agreed with the result obtained in rats by Miller et al. [30].

Vitamin B6 is a cofactor in enzymatic reactions involved in tryptophan metabolism, and XA is one of the metabolites. The suboptimal vitamin B6 deficiency could cause accumulation of XA in plasma and urine [22, 31, 32]. PLP is the active form of vitamin B6 in vivo. PLP and XA are usually considered as biomarkers to estimate the vitamin B6 bioavailability in plasma to evaluate vitamin B6 status in organisms. The plasma PLP and the significantly highest level of XA were detected in the −VB6− groups. Correlation analysis also showed that the plasma PLP level was negatively correlated to plasma XA level with a Pearson correlation coefficient of −0.618 (Table 3). So, it indicated that the vitamin B6-deficient mouse model was successfully established, which was in agreement with the prior researches [20, 31, 32].

The vitamin B6 deficiency could slightly reduce lymphocyte proliferation capacity. There was a weak correlation between PLP and SI with a Pearson correlation coefficient of 0.283, agreeing with the study by Willis-Carr that vitamin B6-deficient diet could reduce the number of T lymphocytes due to an impaired immune function in response to ConA stimulation [33]. Cheng et al. reported a relatively weak correlation between plasma PLP level and immune cells, and 100 mg Vitamin B6 daily supplementation did not show more improvement of immune function in severely ill patients than the 50 mg vitamin B6 supplementation daily [34]. Sun also found that more than 700 mg vitamin B6/kg of body weight of vitamin B6 supplementation cannot improve the proliferation capacity of lymphocytes in mice anymore but rather suppressed it [35]. We also found that supplement of vitamin B6 with normal diet doses could significantly recover the lymphocyte proliferation capacity (*P* < 0.05), but the excessive supplementation did not (*P* > 0.05). It indicated that supplementation of vitamin B6 can recover the impaired immunity caused by a short-term vitamin B6 deficiency, but the excessive supplementation might not do much better.

The supplementation of vitamin B6 could affect the differentiation of immature T cells to mature T cells [7, 36], and it could increase immune responsiveness of T cells but not that of B cells [33]. CD3+ plays a role by participating in the assembly and stability of the T cell receptor/CD3 complex on a mature T lymphocyte and transducing the immune signal [37, 38]. Therefore, no significant reduction of CD3 in −VB6− groups indicated that vitamin B6 deficiency would not impair the immune signal transduction ability significantly. However, significant decrease of CD3 in −VB6− group encountering a ConA stimulation indicated that the immune responsiveness of mice reduced (*P* < 0.05) (Figure 3(a)), suggesting that vitamin B6 plays an important role in cell-mediated immunity. Meanwhile, the ratio of CD4/CD8, a marker of immune dysfunction [39], in −VB6− group was significantly less than that in +VB6+ group (*P* < 0.05) (Figure 3(b)). CD4+ plays an important role in regulating immune functions, and CD8+ is mainly responsible for removal of target cells by killing them directly [40]. Therefore, it also suggested that vitamin B6 deficiency caused a decline in immunomodulatory activity encountering a ConA stimulation and a 5-week supplement of vitamin B6 did not significantly recover it (*P* > 0.05). It also indicated that the supplementation of vitamin B6 could recover from the declined immunity caused by a short-term vitamin B6 deficiency but not completely.

CD4+ T-helper cells played an important role in regulating immune functions. It can be induced into two types of T-helper cells: Th1 and Th2, which can activate cytokine IL-2 and IL-4, respectively [41–43]. Besides being produced predominantly by natural killer (NK) and natural killer T (NKT) cells as part of the innate immune response, IFN-*γ* is also produced by CD4+ Th1 and CD8+ cytotoxic T lymphocyte (CTL) and effector T cells once antigen-specific immunity develops [44]. With ConA stimulation, vitamin B6 supplementation with normal diet dose did not recover IL-2 secretion. Excessive vitamin B6 supplementation did not promote the IL-2 secretion much more than normal diet either. It agreed with the report by Doke et al. that vitamin B6-depletion decreased IL-2 secretion and vitamin B6-repletion with a dose of 7.0 mg/100 g diet did not recover its secretion level more than vitamin B6-repletion with dose 0.7 mg/100 g diet [15]. A study conducted in elderly adults also showed that IL-2 production is significantly reduced by vitamin B-6 depletion (61% decrease), while the normal dose of vitamin B6 supplementation could not reverse that change and IL-2 production in only three of the subjects increased by consuming large amounts of vitamin B-6 during the last 4 days of the research [36]. IL-4 production of lymphocytes in vitamin B6 deficient mice was promoted more than that in vitamin B6 normal mice, which exhibited a trend similar to that obtained by Doke et al. [15]. The vitamin B6 deficiency did not change IFN-*γ* production, which was also described in a study by Kjeldby et al. [8]. They found that vitamin B6 deficiency via the feeding of 4-deoxypyridoxine, a potent antagonist of vitamin B6 coenzyme, does not impair the production of IFN-*γ* in mice infected with* Trichinella spiralis *[8]. Collectively, these results demonstrated that normal levels of vitamin B6 are enough to meet the requirement of communication between immune cells, especially in a Th-1 response.

Janus tyrosine kinase/signal transducer and activator of transcription (JAK - STAT) signaling pathway exists in almost all cytokine signaling pathways, through which some cytokines conduct the immunomodulation [18]. Cytokines can induce the signaling cascades simultaneously and there is an antagonistic system to terminate or attenuate the signal to reach a homeostasis. The suppressor of cytokine signaling (SOCS) can suppress the cytokine signal transduction through the JAK/STAT signaling pathway [19]. Marine et al. [20] stated that SOCS-1 may inhibit the differentiation of cells in which IFN-*γ* was expressed or terminate the IFN-*γ* signal transduction. In this study, without ConA stimulation, the transcriptional levels of* SOCS-1* negatively correlated with IFN-*γ* expression levels, with a correlation coefficient of −0.712. After ConA stimulation, it also exhibited a negative correlation between the transcriptional level of* SOCS-1* and IFN-*γ* expression level, with the correlation coefficient of −0.758. Also, the transcriptional levels of* SOCS-1* were positively correlated with the percentage of CD4+ T-helper cells, with the correlation coefficient of 0.651, which might be due to the fact that the IFN-*γ* level showed negative correlation with the percentage of CD4+ T-helper cells, with the correlation coefficient of −0.972. Therefore, it may be concluded that SOCS1 inhibited the differentiation of cells to influence IFN-*γ* expression before encountering an immunostimulation, but it would suppress IFN-*γ* expression levels to terminate its signal transduction after a stimulation, which supported Marine et al.'s hypothesis [20]. Additionally, a positive correlation was exhibited with IFN-*γ* expressed in CD4+ Th1 of the +VB6+ group and −VB6− groups, suggesting that T-bet acted in an upregulation role in cytokines-induced signaling [45].

IFN-*γ*, a potent activator of cell-mediated immunity, could activate the JAK-STAT signaling pathway through interaction with the cytokine receptor, resulting in the induction of SOCS1 and T-bet [46]. SOCS1 can bind to the JAKs using its SH2 domain and inhibit its catalytic activity of inducing STAT1 phosphorylation to inhibit signaling. Nevertheless, T-bet, a member of the T-box family of transcription factors, has been shown to promote TH1 development and IFN-*γ* production [45]. Although T-bet can be expressed in all these cell types, it was more prominent in CD4+ as it was required for control of IFN-*γ* production in CD4+ cells, which is not needed in CD8 cells [45, 46]. Hence, it is understandable that the CD8+ cells did not change much; meanwhile the IFN-*γ* increased after ConA stimulation (Figures 3(c) and 4(c)). Taken together, the results showed that vitamin B6 deficiency weakened the immune response through affecting T lymphocyte differentiation and proliferation and IFN-*γ* expression and further affecting* SOCS1* and* T-bet *transcription, which were involved in JAK/STAT signaling pathway.

## 5. Conclusions

This study successfully established a vitamin B6 deficiency and recovery using BALB/c mouse model. Results demonstrated that vitamin B6 deficiency influenced the immune system in three ways: (1) downregulation of SOCS-1 expression, as well as upregulation of T-bet expression, (2) suppression of T lymphocyte differentiation, and (3) decreased levels of IL-2 secretion and increased levels of IL-4 secretion. Appropriate supplementation of vitamin B6 could recover the impaired immunity caused by a short-term vitamin B6 deficiency. Further studies are needed to deeply investigate the effects of vitamin B6 deficiency on JAK/STAT signaling pathway.

## Figures and Tables

**Table 1:** Diet composition of vitamin B6 deficient mouse model.

Diet composition		Mineral/Kg diet		Vitamin/Kg diet	
Sugar	40%	MgCl·6H_2_O	17 g	Vitamin A	14000 IU
Casein	20%	KCl	5 g	Vitamin D	1500 IU
Starch	18%	NaCl	6 g	Vitamin E	1200 IU
*α*-Cellulose	8%	FeCl_3_	579 mg	Vitamin B1	13 mg
Oil	5%	MnCl_4_·4H_2_O	270 mg	Vitamin B2	12 mg
Mineral	3.5%	CuSO_4_·5H_2_O	40 mg	Vitamin B6	0/12/120 mg^*∗*^
Vitamin	1%	ZnCl_2_	63 mg	Folic acid	6.0 mg
Methionine	0.30%	Sn	0.2 mg	Niacin	45 mg
Choline Bitartrate	0.20%			Pantothenic acid	17 mg
				Biotin	0.10 mg
				Vitamin K	3.0 mg

^*∗*^Vitamin B6 was given as pyridoxine (PN) and its content was different in different diets. The diet was strictly defined as Non-VB6 Diet, Normal-VB6 Diet, and Excessive-VB6 Diet, which contained 0, 12 mg, and 120 mg of vitamin B6 per kg diet, respectively. The Excessive-VB6 Diet was mixed with 10 times vitamin B6 of the daily recommended intake.

**Table 2:** Plasma xanthurenic acid (XA) and pyridoxal-5′-phosphate (PLP) concentrations at the end of the vitamin B6-deficiency stage (−VB6−) and the other three stages of vitamin B6: normal (+VB6+), recovery (−VB6+), and excess (−VB6++).

	Plasma XA (ng/mL)	Plasma PLP (pg/mL)
+VB6+	140.51 ± 12.08^a^	205.18 ± 182.26^a^
−VB6−	196.61 ± 28.07^b^	ND
−VB6+	141.42 ± 20.61^a^	329.56 ± 175.00^a^
−VB6++	127.90 ± 25.18^a^	341.56 ± 193.69^a^

Data represent the mean ± SD of seven replicates with three independent experiments. Significant difference was indicated with different letters (*P* < 0.05).

**(a):** 

	PLP	XA	SI	CD4	CD8	CD4/CD8	IL-2	IL-4	IFN-*γ*	SOCS-1	T-bet
PLP	1.000										
XA	−0.618	1.000									
SI	0.284	−0.430	1.000								
CD4	−0.684	−0.182	−0.105	1.000							
CD8	0.684	0.182	0.105	−1.000^*∗∗*^	1.000						
CD4/CD8	−0.551	−0.275	−0.163	0.988^*∗*^	−0.988^*∗*^	1.000					
IL-2	−0.767	0.970^*∗*^	−0.530	0.052	−0.052	−0.037	1.000				
IL-4	0.741	0.005	0.311	−0.974^*∗*^	0.974^*∗*^	−0.962^*∗*^	−0.236	1.000			
IFN-*γ*	0.968^*∗*^	−0.513	0.034	−0.666	0.666	−0.552	−0.647	0.706	1.000		
SOCS-1	−0.828	0.940	−0.533	0.158	−0.158	0.069	0.994^*∗∗*^	−0.337	−0.712	1.000	
T-bet	−0.183	−0.630	0.010	0.866	−0.866	0.919	−0.423	−0.779	−0.217	−0.327	1.000

**(b):** 

	PLP	XA	SI	ΔCD4	ΔCD8	ΔCD4/ΔCD8	ΔIL-2	ΔIL-4	ΔIFN-*γ*	ΔSOCS-1	ΔT-bet
PLP	1.000										
XA	−0.618	1.000									
SI	0.284	−0.430	1.000								
ΔCD4	−0.905	0.235	−0.221	1.000							
ΔCD8	0.905	−0.235	0.221	−1.000^*∗∗*^	1.000						
ΔCD4/ΔCD8	−0.845	0.139	−0.281	0.989^*∗*^	−0.989^*∗*^	1.000					
ΔIL-2	0.391	−0.730	−0.300	−0.024	0.024	0.123	1.000				
ΔIL-4	0.670	0.146	0.149	−0.922	0.922	−0.959^*∗*^	−0.323	1.000			
ΔIFN-*γ*	0.839	−0.180	0.399	−0.972^*∗*^	0.972^*∗*^	−0.992^*∗∗*^	−0.166	0.938	1.000		
ΔSOCS-1	−0.287	−0.470	−0.256	0.651	−0.651	0.752	0.732	−0.881	−0.758	1.000	
ΔT-bet	−0.674	0.787	−0.858	0.478	−0.478	0.466	−0.170	−0.236	−0.552	0.084	1.000

The correlations were calculated as Pearson's correlation coefficient matrix. CD, cluster of differentiation; IFN-*γ*, interferon gamma; IL-2, interleukin-2; IL-4, interleukin-4; PLP, pyridoxal 5′-phosphate; SOCS-1, suppressor of cytokine signaling 1; T-bet, T-box transcription factor; XA, xanthurenic acid; Δ means the indictor was detected after ConA stimulation.

^*∗*^ *P* < 0.05.

^*∗∗*^ *P* < 0.01.
